# Assessment of Malocclusion and Gender Differences in Orthodontic Treatment Needs Among Adolescents Aged 10-13 Years in Eastern India: A Cross-Sectional Study

**DOI:** 10.7759/cureus.75442

**Published:** 2024-12-10

**Authors:** Vijeta Patri, Gaurav Patri, Nivedita Sahoo

**Affiliations:** 1 Department of Orthodontics, Hi-Tech Dental College and Hospital, Bhubaneswar, IND; 2 Department of Conservative Dentistry and Endodontics, Kalinga Institute of Dental Sciences, Kalinga Institute of Industrial Technology (KIIT) Deemed to be University, Bhubaneswar, IND; 3 Department of Orthodontics and Dentofacial Orthopedics, Kalinga Institute of Dental Sciences, Kalinga Institute of Industrial Technology (KIIT) Deemed to be University, Bhubaneswar, IND

**Keywords:** adolescent, epidemiology, index of orthodontic treatment need, malocclusion, orthodontics, prevalence

## Abstract

Introduction: Malocclusion is a prevalent public health concern among adolescents, impacting both dental health and psychosocial well-being.

Aims and objectives: To assess the prevalence of orthodontic treatment needs among adolescents aged 10-13 years in Bhubaneswar using the dental health component (DHC) and aesthetic component (AC) of the Index of Orthodontic Treatment Need (IOTN) and to analyze gender-based differences.

Methodology: A cross-sectional study was conducted among 450 school-going children aged 10-13 years using cluster sampling across Bhubaneswar. The DHC and AC of the IOTN were used to evaluate treatment needs, and assessments were performed by a single examiner under natural light using standardized tools. Data were analyzed using SPSS Statistics software (IBM Corp., Armonk, NY), and chi-square tests were applied to determine the association between DHC and AC grades and gender, with a significance level of p ≤ 0.05.

Results: The DHC showed 192 (42.67%) children had a definite need for treatment, while the AC indicated 29 (6.4%) children with definite aesthetic needs. Significant gender differences were observed in the AC (p = 0.027), with females exhibiting higher aesthetic concerns. However, no gender difference was found in the DHC (p = 0.876).

Conclusion: The findings highlight a significant prevalence of malocclusion among early adolescents in Bhubaneswar, emphasizing the need for early orthodontic intervention and tailored orthodontic services to address the specific needs of this population.

## Introduction

Oral health constitutes a vital element of overall health and is indispensable for the comprehensive health and well-being of an individual [1]. Dental malocclusions rank as the third most prevalent condition among oral pathologies globally and are widely recognized as a pressing public health issue among younger populations [2]. Malocclusion is not classified as a disease in the strict sense; rather, it represents a morphological variation that may or may not correlate with pathological conditions [3]. Dental afflictions, particularly those such as malocclusion and traumatic dental injuries, not only compromise the functional capacity and aesthetic appeal of an individual but also impact psychological well-being, which may subsequently affect self-esteem, social interactions, and interpersonal relationships, thereby impairing their overall quality of life [4].

In contemporary society, there has been a heightened awareness regarding the significance of dental aesthetics from childhood through adolescence and into early adulthood [3]. Orthodontic interventions are increasingly sought after, largely due to patients’ perceptions concerning the influence of oral health on overall quality of life and available treatment options. Notably, children and adolescents exhibit heightened sensitivity to various factors, such as physical appearance, which may impact their immediate quality of life and psychological growth, ultimately affecting their social competencies and educational outcomes [5].

The incidence of malocclusion varies significantly across nations, age groups, and genders, ranging from as low as 11% to as high as 91% [6]. In a culturally diverse and expansive nation such as India, the prevalence of malocclusion demonstrates substantial disparities across various regions. Contributing factors may include differences in ethnicity, nutritional conditions, religious practices, and dietary patterns. The reported prevalence of malocclusion in distinct regions of India ranges from 20% to 43% [6].

For effective planning of orthodontic preventive measures within a population, epidemiological studies are crucial. Such studies provide insights into the prevalence of various malocclusion types, their etiological factors, the need for orthodontic treatment, and the resources required to deliver these services [1,6]. In the context of evidence-based dentistry, quantitative measures are indispensable for evaluating treatment needs and comparing outcomes against established standards of care. Several indices have been devised to evaluate orthodontic treatment needs and outcomes for both practitioners and patients, ensuring validity and reliability [1]. Despite these advancements, a universally accepted index is yet to emerge. The Index of Orthodontic Treatment Need (IOTN), introduced by Brook and Shaw in 1989, has achieved notable recognition in the UK and parts of Europe [7]. This index prioritizes orthodontic treatment by addressing either dental health or aesthetic considerations. It comprises two main components: the dental health component (DHC), as advocated by the Swedish Medical Board, and the aesthetic component (AC), formulated by Evans and Shaw in 1987 [7,8]. The IOTN classifies malocclusions based on their impact on dental health and perceived aesthetic detriments, thereby identifying individuals most likely to benefit from orthodontic intervention [5].

The deficiency in the literature regarding early adolescence and the lack of focused research on the prevalence and severity of malocclusion during this critical developmental stage are evident in Eastern India. Although studies have been conducted in other parts of India, such as North Karnataka, Himachal Pradesh, and Mysuru, there is a notable gap in data for Eastern India, especially in diverse areas like Bhubaneswar, Odisha [1-6,9,10]. This gap emphasizes the need for localized studies to understand malocclusion prevalence and treatment needs of the study area.

The IOTN was chosen to conduct this study for its comprehensive framework, integrating the DHC and the AC to assess clinical severity and aesthetic impact holistically. Unlike indices such as the Dental Aesthetic Index (DAI), which focuses primarily on aesthetics, or the Peer Assessment Rating (PAR), which measures treatment outcomes, the IOTN provides a balanced evaluation by prioritizing severe clinical features and incorporating aesthetic concerns [5]. By focusing on this underrepresented population and utilizing a robust assessment tool, the study aims to evaluate the prevalence and severity of malocclusion, along with the orthodontic treatment needs of early adolescents aged 10-13 years in Bhubaneswar, Odisha, employing the IOTN, which incorporates both the DHC and the AC. Furthermore, it investigates potential gender-based differences in these treatment needs. The proposed hypothesis suggests no significant differences in orthodontic treatment needs, including the DHC and AC components, among adolescents aged 10-13 years in Bhubaneswar, Odisha, based on gender.

## Materials and methods

Study population

This cross-sectional, community-based study was conducted from October 2023 to March 2024 after obtaining clearance from the institutional ethical committee. This study was conducted amongst school-going children aged 10-13 years from Bhubaneswar, Odisha. A cluster sampling approach has been followed to select the schools from Bhubaneswar based on its assembly constituencies that have been divided into three parts (Bhubaneswar - North, Central, and Ekamra). Two schools, one government and one private, imparting primary and secondary education were selected from each zone by simple random sampling following the lottery method. Dividing schools into assembly constituencies ensured a geographically representative sample of Bhubaneswar and encompassed its diverse socioeconomic, cultural, and educational background. This stratification enhances the study's generalizability by reducing regional biases and including a more varied population. By incorporating both government and private schools, the study reflects a broader range of orthodontic needs, making the findings more applicable to the city's adolescent population as a whole.

Sample size estimation

The sample size for the study was calculated using OpenEpi software (version 3.04) based on parameters derived from previous studies. Key inputs included a 95% confidence interval (CI), 5% significance level (alpha), 20% type 2 error (beta), 80% power, a Z-value of 1.96, and an effect size of 20%. The formula used was:

n = \frac{p (100 - p) z^2}{E^2}.

Here, 𝑛 is the sample size, *p* is the condition's occurrence rate, *E* is the margin of error, and *z* is the value for the confidence level.

A minimum sample size of 450 was determined, and the final study sample included 450 students, with 75 students from each school.

Inclusion criteria

The study included children aged 10-13 years enrolled in the selected schools. Participation was limited to those present on the examination day who provided informed consent from school authorities, parents' consent, and personal assent to participate with the written assurance that their personal data would not be revealed or photographs published without their permission.

Exclusion criteria

Children with a history of prior or ongoing orthodontic treatment, extensive dental caries, or craniofacial anomalies or syndromes were excluded.

Recording the IOTN

To assess normative orthodontic treatment needs, a modified IOTN was applied. A single examiner trained in recording the IOTN index, performed a type III examination, following the American Dental Association guidelines, using a mirror and probe under natural light conditions. The examinations were conducted at school premises with disposable gloves and mouth mirrors. A periodontal probe measured dimensions in millimeters, while the DHC ruler categorized malocclusion grades [1].

The DHC of the IOTN (Table 1) classifies malocclusion into five grades, from grade 1 (no treatment required) to grade 5 (severe need). The most severe occlusal feature for each participant determined their grade [5]. The DHC follows a hierarchical approach, using the MOCDO acronym (missing teeth, overjets, crossbites, displacement of contact points, overbites) to identify the most critical malocclusion trait for clinical application [5,10].

**Table 1:** The dental health component of the Index of Orthodontic Treatment Needs. RCP: retruded contact position; ICP: intercuspal position.

Code	Occlusal traits	Grade 1	Grade 2	Grade 3	Grade 4	Grade 5
a	Overjet	Includes minor contacts; point displacements <1 mm	3.5-6 mm; competent lips	3.5-6 mm	6-9 mm	9 mm and above
b	Reverse overjet	0-1 mm; no	1-3.5 mm		3.5 mm + masticatory or speech difficulties	
c	Crossbite anterior/posterior	<1 mm; discrepancy RCP—ICP	1-2 mm; discrepancy RCP—ICP	2 mm + discrepancy RCP—ICP		
d	Displaced contact points	1-2 mm	2-4 mm		4+ mm = severe	
e	Open bite anterior/posterior	1-2 mm	2-4 mm		4+ mm = severe	
f	Overbite	Up to 3.5 mm; no gingival contact	Complete on gingiva or palate; no trauma		Complete with trauma	
g	Pre- or post-normal occlusion	Grade 2 only (if no other anomalies are present and include up to 1/2 unit discrepancy)				
h	Hypodontia				Less extensive hypodontia; requiring pre-restorative orthodontics or orthodontic space closure to obviate the necessity for prosthesis	Extensive hypodontia with restorative implications (>1 tooth missing in any quadrant); requiring pre-restorative orthodontics
i	Impeded eruption of teeth except third molars					Grade 5 due to crowding, displacement, the presence of supernumerary teeth, retained deciduous teeth, and any pathological cause
j	Posterior lingual crossbite				Grade 4 with no functional occlusal contacts in one or both buccal segments	
k	Reverse overjet (see b)				1-3 mm; recorded masticatory or speech difficulties	3.5 mm + recorded masticatory or speech difficulties
p	Cleft lip/palate craniofacial anomalies					Grade 5 only
s	Submerged deciduous teeth					Grade 5 only
t	Partially erupted, tipped, and impacted against adjacent teeth				Grade 4 only	
x	Presence of supernumerary teeth				Grade 4 only	

The AC of the IOTN evaluated perceived treatment needs. Participants reviewed 10 colored photographs of anterior teeth, illustrating different malocclusion patterns, and selected the image that best matched their dentition. Initially, a mirror was provided for reference and later removed to encourage unbiased selection [1,5].

The AC employs a 10-point scale, where grade 1 represents optimal aesthetics and grade 10 denotes severe aesthetic compromise [5]. It emphasizes overall dental appearance rather than specific morphological features. Black-and-white images minimized bias from gingival or tooth color and hygiene [5]. This component complements the DHC by assessing anterior aesthetic discrepancies through self-assessment [11].

Statistical analysis

Statistical analysis was performed using SPSS software (version 21.0, IBM Corp., Armonk, NY). The chi-square test, with a 5% significance threshold, evaluated gender-based differences in DHC and AC grades and the percentage distribution across categories.

## Results

**Figure 1:**
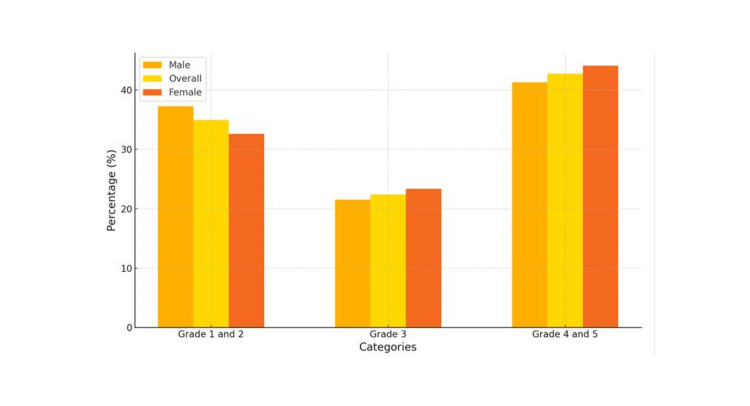
Overall orthodontic treatment needs by category and gender.

Table 2 and Figure 1 present the distribution of DHC grades by gender. The DHC grades range from 1 (indicating no treatment need) to 5 (indicating severe treatment need). Males had a distribution of 14 (6.28%) in grade 1, 69 (30.94%) in grade 2, 48 (21.53%) in grade 3, 71 (31.84)% in grade 4, and 21 (9.42)% in grade 5, with a total count of 223. Females showed similar percentages, with 11 (4.85%) in grade 1, 63 (27.75)% in grade 2, 53 (23.35%) in grade 3, 76 (33.48%) in grade 4, and 24 (10.57%) in grade 5, totaling 227. In examining the distribution of dental health needs across genders, grades 1 and 2, which represent minimal treatment needs, encompass 83 (37.22%) of the male and 74 (32.60%) of the female participants. This indicates a substantial portion of the population has little or no need for orthodontic intervention. Grade 3, which represents a moderate need for treatment, includes 48 (21.53%) males and 53 (23.35%) females. Notably, grades 4 and 5, indicating severe and very severe treatment needs, are more prevalent, encompassing 92 (41.26%) males and 100 (44.05%) females. This trend highlights a significant need for orthodontic intervention within the population. The chi-square test indicated no significant association between the DHC grade and gender (χ² = 1.215, df = 4, p = 0.876). This suggests that the distribution of orthodontic treatment needs is generally uniform between genders, supporting the notion that orthodontic requirements are equally distributed across male and female populations without gender-based disparity.

**Table 2:** Distribution of dental health component grades according to gender. * The chi-square test was done for significance testing. A p-value of ≤0.05 is considered to be significant.

Gender		Grade 1	Grade 2	Grade 3	Grade 4	Grade 5	Total	Value	df	p
Male	Count	14	69	48	71	21	223	1.215	4	0.876
	% within row	6.278%	30.942%	21.525%	31.839%	9.417%	100%			
Female	Count	11	63	53	76	24	227			
	% within row	4.846%	27.753%	23.348%	33.480%	10.573%	100%			

Table 3 presents the distribution of AC grades by gender, where AC grades range from 1 (indicating no or little aesthetic treatment need) to 10 (indicating severe aesthetic treatment need). Among males, 64 (29.22%) fall into both grade 1 and grade 2, 52 (23.74%) in grade 3, 18 (8.22%) in grade 4, and smaller numbers in higher grades: five (2.28%) in grade 5, six (2.74%) in grade 6, three (1.37%) each in grades 7, 8, and 9, and one (0.46%) in grade 10, totaling 219 males. Females show a similar distribution in the lower grades, with 50 (21.65%) in grade 1, 52 (22.51%) in grade 2, and 51 (22.08%) in grade 3, but slightly higher counts in higher grades, with 27 (11.69%) in grade 4, 11 (4.76%) in grade 5, 12 (5.20%) in grade 6, six (2.60%) in grade 7, 14 (6.06%) in grade 8, and four (1.73%) in both grades 9 and 10, totaling 231 females. When assessing the overall distribution, grades 1 to 4, which represent little treatment need, encompass 198 (90.40%) males and 180 (77.93%) females. Moderate treatment need (grades 5 to 7) is slightly more common among females, with 29 (12.56%) compared to 14 (6.39%) males, while definite treatment required (grades 8 to 10) showed a greater prevalence among females, with 22 (9.52%) compared to seven (3.20%) males. This suggests that females tend to report higher aesthetic needs, especially in the upper AC grades. The chi-square test indicated a statistically significant association between the AC grade and gender (χ² = 18.774, df = 9, p = 0.027), signifying a gender-based difference in aesthetic needs distribution, with females more frequently assigned to higher AC grades. This finding highlights a notable gender variation in perceived aesthetic needs within the population, with females showing a greater inclination toward moderate to definite treatment needs, reflecting potential variations in self-perception or aesthetic awareness.

**Table 3:** Distribution of aesthetic component (AC) grades according to gender. * The chi-square test was done for significance testing. A p-value of ≤0.05 is considered to be significant.

Gender	Code AC	1	2	3	4	5	6	7	8	9	10	Total	Value	df	p
Male	Count	64	64	52	18	5	6	3	3	3	1	219	18.774	9	0.027*
	% within row	29.224%	29.224%	23.744%	8.219%	2.283%	2.740%	1.370%	1.370%	1.370%	0.457%	100.000%			
Female	Count	50	52	51	27	11	12	6	14	4	4	231			
	% within row	21.645%	22.511%	22.078%	11.688%	4.762%	5.195%	2.597%	6.061%	1.732%	1.732%	100.000%			

**Figure 2:**
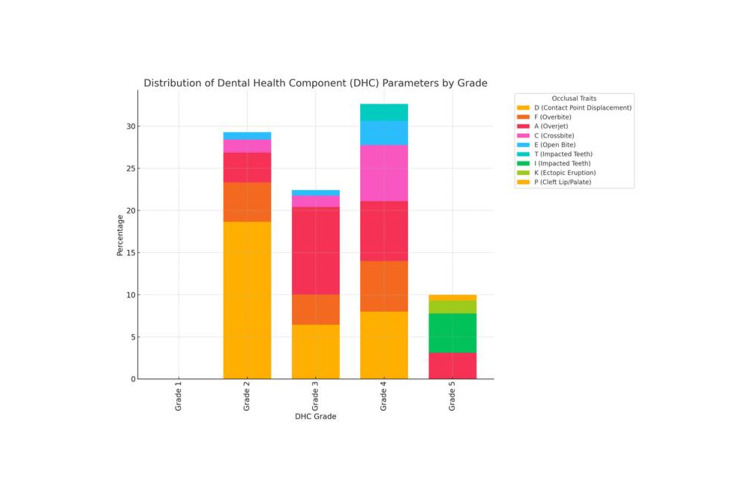
Dental health component (DHC) parameters (occlusal traits). This is a stacked bar graph showing the distribution of DHC parameters by grade. Each bar represents a DHC grade, with different occlusal traits (such as contact point displacement, overbite, overjet, etc.) displayed as segments within each bar, indicating their percentage within each grade.

Figure 2 presents the distribution of DHC parameters, categorized by specific occlusal traits across five grades, each indicating the severity of orthodontic treatment needs. In grade 1 (no need for treatment), 25 (5.56%) of the population displays extremely minor occlusal deviations. Grade 2 (little treatment need) represents 132 (29.34%) of the population, primarily due to contact point displacement (D) in 84 (18.66%), followed by increased overjet (A) in 16 (3.55%), increased overbite (F) in 21 (4.66%), anterior or posterior crossbite (C) in seven (1.55%), and open bite (E) in four (0.88%). Grade 3 (moderate treatment need) includes 101 (22.44%) individuals, with increased overjet (A) in 47 (10.44%), contact point displacement (D) in 29 (6.44%), overbite (F) in 16 (3.55%), crossbite (C) in six (1.33%), and open bite (E) in three (0.66%). Grade 4 (severe treatment need) has the highest prevalence, accounting for 147 (32.67%) of the population. Here, contact point displacement (D) is again predominant in 36 (8.0%), followed by increased overjet (A) in 32 (7.11%), crossbite (C) in 30 (6.66%), overbite (F) in 27 (6.0%), open bite (E) in 13 (2.88%), and impacted teeth (T) in nine (2.0%). Finally, grade 5 (very severe treatment need) comprises 45 (10.0%) of the population, with impacted teeth (I) leading in 21 (4.66%), followed by overjet (A) in 14 (3.11%), ectopic eruption (K) in seven (1.55%), and cleft lip/palate (P) in three (0.66%). This distribution emphasizes that grades 4 and 5, indicating higher treatment needs, collectively represent a significant portion of the sample, with contact point displacement, overjet, and impacted teeth being the most common contributing factors to severe malocclusion.

## Discussion

This study uniquely examines orthodontic treatment needs among early adolescents aged 10-13 years in Bhubaneswar, Odisha, utilizing the IOTN. Unlike prior research conducted in regions such as Bengaluru [5], Mysuru [1], North Karnataka [9], and Himachal Pradesh [6], which primarily focused on older children or adolescents aged 12 and above, no studies have explored this younger demographic in Eastern India, specifically Bhubaneswar. Additionally, existing literature reveals conflicting findings regarding gender-based differences in orthodontic treatment requirements [5,10]. By targeting this underrepresented and critical developmental age group, the study offers valuable insights into the prevalence and severity of malocclusion. It also investigates potential gender disparities through the application of both the DHC and AC of the IOTN.

The World Health Organization (WHO) classifies early adolescence as ages 10-13 years [6]. This range captures the onset of puberty and significant physical, cognitive, and emotional development, including the eruption of permanent teeth, making it an ideal time to assess and address orthodontic needs [5]. This age group exhibits a high prevalence of malocclusion, which, if untreated, can lead to long-term dental and psychosocial issues [6]. Early identification allows for timely interventions thus improving long-term outcomes [8]. This age group was thus included in the study.

In this study, care was taken to keep the percentage of the gender difference equal. The percentage of gender difference was kept equal to evaluate the treatment needs and facilitate direct comparisons between male and female participants. Additionally, it eliminates any potential gender bias in the results and enhances the statistical validity of the findings. Gender difference was also kept to assess the self‑perception among the males or females toward the malocclusion [6].

The DHC of the IOTN categorizes the severity of malocclusion into five grades, grouped into three main categories to assess orthodontic treatment requirements: no or minimal need (grades 1 and 2), moderate need (grade 3), and severe or definite need (grades 4 and 5) [1,10]. Similarly, the AC of the IOTN evaluates overall dental aesthetics rather than individual morphological features. Grades 1-4 signify minimal or no treatment need, grades 5-7 indicate moderate or borderline need, and grades 8-10 reflect a clear requirement for treatment [5]. In this study, results are discussed following this classification to maintain consistency and enable meaningful comparisons with findings on orthodontic treatment needs in other populations and studies.

Our study estimates that 192 (42.67%) of early adolescents in Bhubaneswar, Odisha, require orthodontic treatment (grades 4 and 5), as evaluated by the DHC of the IOTN (Table 2). These findings align closely with previous studies reporting similar prevalence rates of 49.37% in the Punjab population [6], 49.3% in the North Karnataka population [9], 47.9% in the Malaysian population [12], 42% in the Maltese and Gozitan populations [13], 38.8% in the Turkish population [14], and 37.55% in the Nalagarh population [6]. Such consistency may be attributed to the standardized methodology employed and probable similarities in demographic, socioeconomic, and cultural factors across these populations.

In contrast, our findings differ significantly from those reported in other regions: 65% in the Norwegian population [15], 63.4% in the Victorian population [16], 50% in Trivandrum children [17], 30.4% in the Irish population [18], 29% in Kenya [19], 28.8% in the Udupi population [20], 28% in the Canadian population [21], 28% in the Kuwaiti population [22], 23.5% in Spanish children [23], 22% in the Japanese population [24], 22% in Tanzanian children [25], 20.42% in Shimla [26], 13% in Nigerian children [6], 12.5% in Himachal Pradesh [3], 9.0% in the Tehran population [7], and 6.0% in schoolchildren from Yemen [27]. These discrepancies likely reflect variations in population characteristics, sample size, selection criteria, environmental and lifestyle factors, as well as differing levels of awareness and attitudes towards orthodontic treatment.

The treatment needs of males and females were evaluated in this study (Table 2). The DHC of the IOTN demonstrated no statistically significant variation between males and females (p = 0.876) assessed by the chi‑square test. This finding aligns with those reported in previous studies [1,5,6,10,23]. This consistency across multiple studies strengthens the validity of the current study's findings. This also suggests that this may be a common trend in the Indian population being studied. It is possible that the biological and behavioral factors influencing malocclusion are similar for both genders in the studied population, contributing to similar results.

An analysis of the distribution of DHC occlusal traits (Figure 2) in the study sample showed that contact point displacement was the most commonly found occlusal trait followed by increased overjet. Cleft lip and palate were the least common occlusal traits encountered. Chaitra et al. [9], in a north Karnataka population, and Singh et al. [6], in a Nalagarh population, also found contact point displacement to be the dominant occlusal trait. The dominance of contact point displacement can be attributed to a combination of anatomical, cultural, genetic, and environmental factors, as well as the methodologies employed in clinical assessments [6,8]. To generalize, contact point displacement seems to be the dominant occlusal trait in the Indian population.

**Figure 3:**
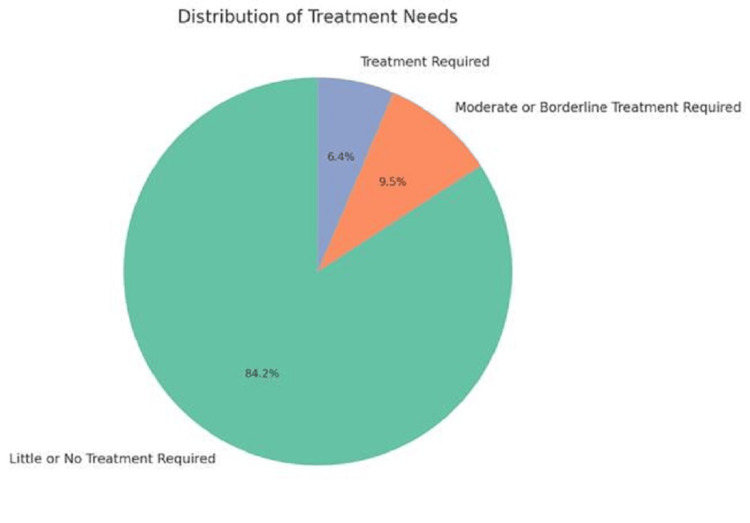
Distribution of treatment needs compiled from the data in Table 3, on the basis of IOTN AC categorization. IOTN: Index of Orthodontic Treatment Need; AC: aesthetic component.

In the current study, among 450 students, based on the AC, 379 (84.2%) exhibited no or little need for orthodontic treatment, while 42 (9.5%) demonstrated a moderate need, and 29 (6.4%) required definitive treatment (Figure 3). These results align closely with several previous studies. For instance, one study observed 6.4% with a definitive need for treatment [5], while others reported similar findings, with 7% [6], 4.4% [28], 4.95% [6], 4.5% [29], and 4.8% [14] requiring definite treatment in their respective cohorts. However, the present findings differ from studies that reported higher percentages of students with a definitive need for treatment. For example, some studies identified 16.1% [30], 21.5% [31], 36% [32], 32.8% [2], 35.6% [1], and even 81.7% [33] requiring treatment. These discrepancies highlight the variation in treatment needs across different populations and methodologies used in the studies.

The AC of the IOTN revealed a statistically significant difference between males and females (p = 0.027) assessed by the chi‑square test (Table 3). Females show more perception toward malocclusion on aesthetic grounds as compared to males. This was in agreement with studies where it was observed that men appeared to be less concerned with the appearance of their teeth, even when observable aesthetic defects were present, whereas females showed concern over minor discrepancies [1,6,10].

Cultural and socioeconomic factors may significantly influence an individual’s judgment regarding dental aesthetics. Research suggests that while subjects are generally capable of making objective evaluations of their teeth, there exists considerable variability in what is deemed acceptable [9]. Some children found the concept of AC challenging to understand, often attempting to match their dentition precisely with the photographs provided, focusing on specific morphological traits. This difficulty was particularly evident in children with conditions such as fractured incisors, congenitally absent bilateral lateral incisors, or peg-shaped lateral incisors, who struggled to select photographs that accurately represented their degree of dental attractiveness [9].

Based on the study results, the null hypothesis is partially upheld. It is supported by the DHC, indicating no gender differences in dental health needs. However, it is rejected for the AC, as significant gender differences were observed in aesthetic perceptions and treatment requirements.

While this study provides valuable insights into the orthodontic treatment needs of adolescents in Bhubaneswar, certain limitations must be acknowledged to contextualize the findings. This study is limited to school-going adolescents in Bhubaneswar, restricting generalizability. Its cross-sectional design precludes longitudinal analysis, and self-assessment for aesthetics may introduce bias. Factors like socioeconomic status, diet, oral hygiene, and environmental or genetic influences were not considered, and the potential subjectivity of a single examiner in evaluations remains a concern.

Future research should broaden its geographic scope to explore malocclusion prevalence across diverse regions of India and include a wider range of populations with varying dental conditions and backgrounds for a more comprehensive understanding. Longitudinal studies are needed to track changes in orthodontic needs and perceptions over developmental stages. Incorporating multiple examiners could enhance reliability, while studies on early orthodontic interventions could provide valuable insights into their long-term dental and psychosocial benefits.

## Conclusions

This study among adolescents in the Bhubaneswar population highlights a significant gap between clinical orthodontic treatment needs (192, 42.67%) and aesthetic self-awareness (29, 6.4%), underscoring the importance of raising awareness about malocclusion severity. Gender analysis revealed that females are more concerned with aesthetics, influenced by cultural and psychological factors, though no significant difference was found in clinical needs. Early detection and intervention are crucial to improving oral health, self-esteem, and overall quality of life in this population. Contact point displacement and overjet were identified as the primary malocclusion factors, emphasizing the need for targeted educational and clinical strategies to address the orthodontic needs of Bhubaneswar adolescents effectively.

Finally, to translate these findings into actions, public health initiatives like school-based dental screenings can aid in early malocclusion detection. Educational campaigns for students, parents, and educators should raise awareness about its impact on self-esteem and quality of life. Subsidized orthodontic care in underserved areas can enhance treatment access while integrating malocclusion awareness into school health curriculums and collaborating with dental health organizations can strengthen early intervention. Highlighting the psychosocial and clinical benefits of timely orthodontic care can foster community engagement and policy support, improving oral health outcomes for adolescents.
